# An Insecticidal Compound Produced by an Insect-Pathogenic Bacterium Suppresses Host Defenses through Phenoloxidase Inhibition

**DOI:** 10.3390/molecules191220913

**Published:** 2014-12-12

**Authors:** Ihsan Ullah, Abdul Latif Khan, Liaqat Ali, Abdur Rahim Khan, Muhammad Waqas, In-Jung Lee, Jae-Ho Shin

**Affiliations:** 1 School of Applied Biosciences, College of Agriculture and Life Sciences, Kyungpook National University, Daegu 702-701, Korea; E-Mails: ihsanknu@gmail.com (I.U.); rahimkhan_84@yahoo.com (A.R.K.); agronomist89@gmail.com (M.W.); ijlee@knu.ac.kr (I.-J.L.); 2 Department of Biological Science and Chemistry, University of Nizwa, Nizwa 616, Oman; E-Mails: latifepm78@yahoo.co.uk (A.L.K.); malikhejric@unizwa.edu.om (L.A.)

**Keywords:** insecticidal, compound, phthalic acid, antibacterial, antifungal, antioxidant

## Abstract

A bioassay-guided column chromatographic strategy was adopted in the present study to fractionate the culture extract of *Photorhabdus temperata* M1021 to identify potential insecticidal and antimicrobial compounds. An ethyl acetate (EtOAc) culture extract of *P. temperata* was assayed against *Galleria mellonella* larvae through intra-hemocoel injection and exhibited 100% insect mortality within 60 h. The EtOAc fraction and an isolated compound exhibited phenoloxidase (PO) inhibition of up to 60% and 63%, respectively. The compound was identified as 1,2-benzenedicarboxylic acid (phthalic acid, PA) by gas chromatography-mass spectrometry and nuclear magnetic resonance. PA exhibited insecticidal activity against *G. mellonella* in a dose-dependent manner, and 100% insect mortality was observed at 108 h after injection of 1 M PA. In a PO inhibition assay, 0.5 and 1 M concentrations of PA were found to inhibit PO activity by 74% and 82%, respectively; and in a melanotic nodule formation assay, nodule formation was significantly inhibited (27 and 10 nodules) by PA (0.5 and 1 M, respectively). PA was furthermore found to have substantial antioxidant activity and maximum antioxidant activity was 64.7% for 0.5 M PA as compare to control. Antibacterial activity was assessed by The MIC values ranged from 0.1 M to 0.5 M of PA. This study reports a multifunctional PA, a potential insecticidal agent, could a factor of insect mortality along with other toxins produced by *P. temperata* M1021.

## 1. Introduction

The genus *Photorhabdus* consists of nematode-symbiotic, entomophagous *Enterobacteria* that follow a complex dual-phase life cycle involving mutualistic associations with *Heterorhabditis* nematodes as well as pathogenic interactions with insects [1]. In order to combine symbiosis and pathogenicity, *Photorhabdus* has to produce an array of metabolites that can facilitate both interactions. The *Photorhabdus* genome reveals that more than 50% of the genes in the *Photorhabdus* gene pool are distinct from those of *Escherichia coli* (commonly used as a model system), suggesting that a large number of novel genes are involved in the pathogenicity and symbiosis of these organisms [2]. Moreover, genome analyses of *Photorhabdus* spp. [3,4] reveal that nearly 6% of the genome is dedicated to genes predicted to be involved in the production of secondary metabolites [4]. This proportion is greater than the 3.8% observed in *Streptomyces*, the model organism for secondary metabolite production [4,5]. Thus, there is significant potential for novel bioactive molecule discovery in *Photorhabdus* [5].

Despite the fact that *Photorhabdus* spp. are nematode-symbiotic, these bacteria can be grown on media independently of their nematode hosts, after which they still induce significant insect mortality [6]. *Photorhabdus* bacteria are known to secrete a wide variety of metabolic compounds into the culture medium, including lipases, proteases, antibiotics, lipopolysaccharides, and a number of other secondary metabolites [2,7,8]. The secondary metabolites produced by *Photorhabdus* bacteria are active against a wide range of insects as well as against microbial pathogens of animals and plants, including bacteria and fungi [9,10]. Physiological analyses have revealed that the pathogenicity of these toxins to insects occurs via the suppression of immune responses in the insect host [9,10]. This immune response suppression is facilitated by a number of processes, including induction of hemolysis, degradation of antimicrobial peptides, inhibition of eicosanoid biosynthesis, and suppression of prophenoloxidase (proPO) activation [11]. The phenoloxidase (PO) immune response in insects is an immediate reaction to microbe invasion [12], and proceeds via activation of the PO enzyme, initially present as an inactive precursor (proPO) in the hemolymph plasma, initiated after recognition of invading microbes [11]. After activation, PO catalyzes the conversion of mono- and di-phenolic substrates to quinones, which are subsequently converted to melanin that is deposited at wound sites, forming nodules. These nodules seal the wounds and prevent microbes from entering and spreading throughout the insect body cavity [9,13,14,15]. Pathogens, however, have evolved to interact with the PO system to successfully infect their hosts [10]. Numerous compounds extracted from *Photorhabdus* spp. have been reported to overcome the immune barrier of insects by interfering with the insect PO system [15], resulting in a rapid reduction in hemolymph PO activity and the suppression of the host encapsulation response [9,10].

The metabolites produced by *Photorhabdus* bacteria also exhibit antimicrobial and antioxidant activities, which serve to prevent the growth of competing microorganisms and putrefaction of the nematode-infected insect cadavers [16]. This is important for the successful completion of the life cycle of the nematode parasite and for symbiont transmission to the nematode’s progeny [6]. In culture medium, *Photorhabdus* spp. commonly produce secondary metabolites with insecticidal, antioxidant, and antibiotic properties [10]. These metabolites include stilbene derivatives, anthraquinone derivatives, genistein, furan derivatives, phenol derivatives and glidobactin/luminmycin derivatives [9,17]. In the present study, we aimed to identify one or more new multifunctional, metabolite(s) in a culture extract of *Photorhabdus temperata* M1021, which would exhibit PO inhibition as well as antioxidant and antimicrobial activities. The culture extract was processed using column chromatography and a bioassay-guided isolation yielded a single purified compound that exhibits inhibitory activities against cellular immune responses via PO inhibition. The compound was identified using gas chromatography-mass spectrometry (GC-MS) and nuclear magnetic resonance (NMR) analyses. The identified compound was analyzed for inhibitory effects on PO activity and nodule formation in *Galleria mellonella* larvae, and the antimicrobial and antioxidant activities of the compound were also determined. From the results of this investigation, the isolated compound was characterized as having multiple functions, including immune response suppression via PO inhibition, as well as antimicrobial and antioxidant functions.

## 2. Results and Discussion

### 2.1. Insecticidal Effects of Metabolite Fractions

**Figure 1:**
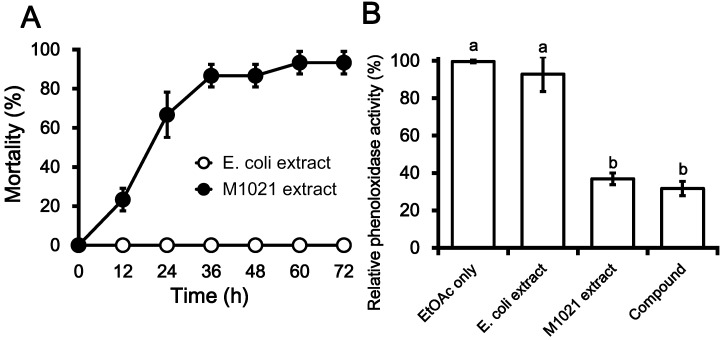
Insecticidal activity due to immune suppression by bioactive extracts of *Photorhabdus temperata* M1021. (**A**) Rate of mortality in *Galleria mellonella* larvae due to the intra-hemocoel injection of the EtOAc extract of *P. temperata* M1021, in a comparison with the EtOAc extract of *E. coli* DH5α used as control; (**B**) Phenoloxidase inhibition by EtOAc extract of *P. temperata* M1021 and purified compound from the extract in a comparison with EtOAc only and EtOAc extract of DH5α used as dual control. The resultant values are the averages of three replications (*n* = 3), each with ten larvae per repeat. The error bars represent mean ± SD of the three repeats. The different letters *i.e*., a and b above the error bars indicate significant differences among each other with *p <* 0.05 as determined by Duncan’s multiple range test (DMRT).

Members of the *Photorhabdus* genus are fascinating entomopathogenic bacteria that have been studied extensively for their insecticidal potential. Several studies have described the insecticidal and antimicrobial properties of these organisms. In the present study, an initial insecticidal bioassay was performed by injecting 10 µL ethyl acetate (EtOAc) extract of *P. temperata* M1021 into the hemocoel of fifth instar *G. mellonella* larvae to assess the insecticidal activity of the extract. Up to >20% of larvae were killed by intra-hemocoelic injections of EtOAc in the first 12 h in a comparison with control-injected larvae, and insect mortality increased to 70%, 90% and 100% after 24, 36, and 60 h, respectively (Figure 1A). These findings are in agreement with those of Jang *et al.* and Salvadori *et al.* [18,19] and suggest that the extracellular metabolites produced by *Photorhabdus* bacteria are toxic towards a diverse group of insects. A culture extract of *Photorhabdus* spp. was reported to have significant insecticidal activity (76%‒83%) 48 h after treatment, and a culture extract of *X. nematophila* was shown to kill 83% of *Luciaphorus perniciosus* mites within 72 h [20,21]. The findings of these studies support the hypothesis that *Photorhabdus* bacteria can produce a large arsenal of insecticidal toxins in culture broth that arrest the immune response of insect larvae, leading to septicemia and therefore death of the insect host.

The bioactive EtOAc extract was further assayed for PO inhibitory effects in *G. mellonella* larvae, and was found to inhibit PO activity by up to 65% in comparison to two controls (EtOAc only and an EtOAc culture extract of *E. coli* DH5α; Figure 1B). Culture extract of *Photorhabdus* analyzed by Seo *et al.* [9] was found to be a potent biological agent against insects, and it was later shown that this insecticidal effect is due to the suppression of insect immune responses by PO inhibition [22]. The PO cascade is an important component of the insect immune system, and inhibition of this cascade triggers various biochemical reactions leading to death in a variety of insects [10]. Early studies suggest that secondary metabolites purified from the extract of *Photorhabdus* spp. can inhibit immune responses, which is important for hemocyte nodulation [9]. Such properties enable *Photorhabdus* spp. to evade the cellular immune response in insects resulting in insect death [10,23].

The bioactive EtOAc fraction from *P. temperata* was fractionated on a silica gel column and a single bioactive sub-fraction was eluted with a methanol (MeOH) and EtOAc (100:0; *v*/*v*) solvent system (Figure S1). The eluted sub-fraction was further subjected to high performance liquid chromatography (HPLC; C_18_ column), which allowed for the purification of compound **1**. EtOAc extract and compound **1** were assessed for PO inhibitory effects in *G. mellonella*, and it was found that the both treatments inhibited PO activity by up to 70% in the larvae relative to two controls (EtOAc alone and EtOAc culture extract of *E. coli*; Figure 1B). PO is a conserved component of the insect immune system that generates cytotoxic intermediates and causes the formation of melanin, which in turn encircles the wound and/or infection [9,10]. The findings presented here suggest that *Photorhabdus* bacteria synthesize one or more compound(s) that not only contribute to the pathogenicity of the bacteria [10], but that allow the organisms to overcome insect immune defenses by suppression of host PO activity [5]. Culture extracts of other nematode-associated bacterial pathogens of insects have also been shown to inhibit PO [24,25]. It was recently reported that the *Microplitis demolitor* bracovirus carried by the *M. demolitor* wasp produces a toxin which inhibits the PO cascade [26], suggesting that suppression of the PO pathway as a means for insect immune response evasion is a common strategy also adopted by insect-specific pathogens other than bacteria.

**Figure 2:**
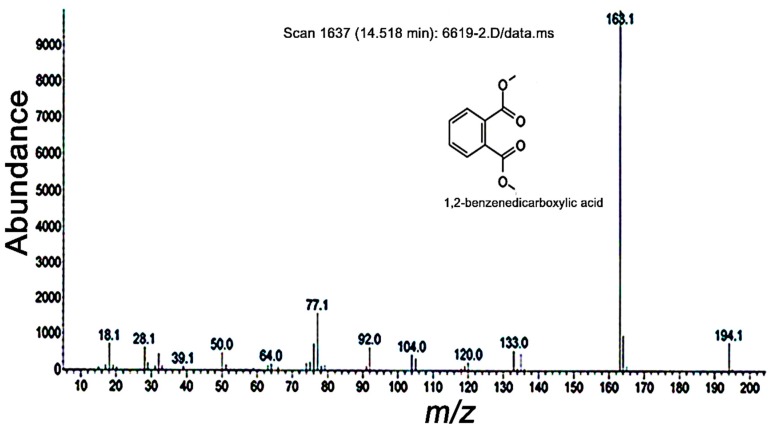
GC-MS analysis of the structure of compound 1 extracted from *Photorhabdus temperata* M1021. The GC-MS chromatogram was obtained by injection of 100 µL of a purified sample onto the column using a 10:1 split injection mode. The oven temperature was initially held at 100 °C for 3 min, raised to 300 °C for 5 min, and finally held at 300 °C for 48 min.

GC-MS analysis of the purified compound **1** (Figure 2) suggests that the isolated compound is dimethyl ester of phthalic acid (PA). This chemical identification was further supported by NMR data (^1^H-NMR, CDCl_3_, 500 MHz). The presence of molecular ion peak M^+^ at *m/z* 194 along with the major fragment at *m/z* 163 (M^+^-H) as the base peak indicated the presence of methoxy group in the molecule. Further fragments at *m/z* 135, 133, 119.9, 104, 92, and 76 were indicative of the presence of two dimethoxy carboxylate groups on the benzene ring. The ortho-position of these groups was further confirmed by NMR spectra. The ^1^H-NMR spectrum exhibited two signals (a doublet of doublets with coupling constants of 6.4 and 2.0) characteristic of a di-substituted benzene ring in the region δ 7.25–7.36 (Figure S2). In agreement with the ^1^H-NMR spectrum, three signals of double intensity also appeared in the proton-decoupled ^13^C-NMR spectrum at δ 124.9, 131.4, and 136.3, corresponding to four sp2 methine carbons (C-3 to C-6) and two carbonyl-bearing quaternary carbons (C-1 and C-2). Two quaternary carbons (C-7 and C-8) of the carboxylic groups appeared at δ 165.7 (Figure S3). All of the above mentioned findings confirmed the purified constituent to be dimethyl ester of 1,2-benzenedicarboxylic acid, commonly known as phthalic acid (PA).

### 2.2. Inhibition PO Activity and Nodule Formation by PA

**Figure 3:**
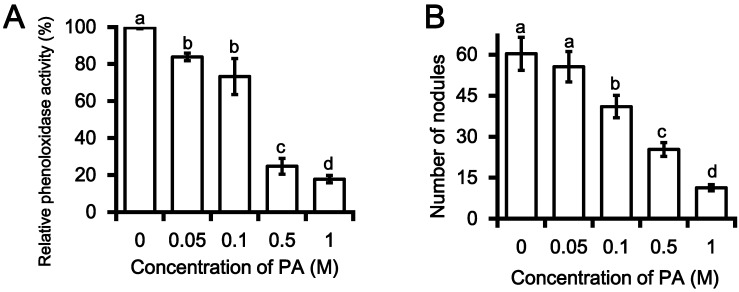
Insecticidal properties of phthalic acid (PA) extracted from *Photorhabdus temperata* M1021. (**A**) Phenol oxidase inhibition by PA in a dose dependent manner in a comparison with treatment of MeOH only, used as control; (**B**) Inhibition of nodule formation in the larvae of *G. mellonella* in the result of PA treatment in a concentration dependent manner as compare to control. *E. coli* DH5α cell (2 × 10^6^) were used as a positive control. The resultant values are the averages of three replications (*n* = 3), each with ten larvae per repeat. The error bars represent mean ± SD of the three repeats. Different letters *i.e*., a, b, c and d over the error bars indicate significant differences at *p <* 0.05 levels as estimated by Duncan’s multiple range test (DMRT).

In the current study, PA was tested for its inhibitory effects on immune-associated characteristics and was found to significantly inhibit PO catalytic activity in a dose-dependent manner. Several PA concentrations ranging from 0.05‒1 M were assessed for inhibitory effects on hemocyte nodulation in response to bacterial challenge. Compared with a control, 0.05 M PA resulted in 18% PO inhibition in *G. mellonella*, while 0.5 M and 1 M PA inhibited PO activity by 74% and 82%, respectively (Figure 3A). The half-maximal inhibitory concentration (IC_50_) value of PO inhibition for PA was calculated to be 0.29 ± 0.01 M.

A number of compounds extracted from *Photorhabdus* spp. have been reported to hamper the PO cascade in insects and thus overcome the immune barrier, leading to insect death [22]. Previous studies [9,10,23] have reported on the identification of various compounds in culture extracts of *Photorhabdus*, including 1,3-dihydroxy-2-(isopropyl)-5-(2-phenylethenyl) benzene, benzylideneacetone, proline-tyrosine, acetylated phenylalanine-glycine-valine indole, oxindole, *cis*-cyclo-PY, and *p*-hydroxyphenyl propionic acid. These compounds exhibited significant insecticidal activity towards a diverse group of larvae, including *Manduca* *sexta*, *Plutella xylostella*, *Salix exigua*, and *G. mellonella*, and this insecticidal activity was attributed to immune response suppression via PO inhibition.

PO catalyzes the melanogenesis reaction that involves the conversion of phenols to quinones. These quinones subsequently polymerize to form melanin that encapsulates the parasitoid by nodule formation, thereby providing a defense against foreign pathogens [9,10,27]. Since nodule formation is a product of PO activity [5], the potential inhibition of nodule formation by PA was assessed in a dose-response experiment. PA was found to inhibit nodule formation at relatively high concentrations: 0.1 M PA significantly (*p* < 0.05) reduced the number of nodules compared 0 M PA, and further decreases in nodule number were observed with increasing PA concentration; 0.5 and 1 M PA resulted in substantial inhibition of nodule formation indicative of a collapse of the insect immune system eventually leading to insect death (Figure 3B). PA is known to inhibit two key immune defenses of insects: PO activity and melanotic nodule formation [22]. Upon closer investigation into the insect immune system, we discovered that these two host defenses targeted by PA are functionally linked: PO activation is required for the production of melanotic nodules [10].

Cerenius *et al.* [28] reported that many factors are involved in the activation of the PO cascade such as bacterial peptidoglycan. The activated PO subsequently catalyzes the conversion of mono- and di-phenolic substrates to quinones, which are then converted to melanin. A number of studies have also shown that melanin deposition provides a defense against bacteria and multicellular parasites [5,10]. Hemocyte nodule formation is a biphasic process consisting of hemocyte aggregation and melanization phases [9]. PO activity is required for melanization [29], and inhibition of PO by bacterial metabolite(s) thus results in a suppression of hemocyte nodule formation in response to bacterial challenge [9,10]. The findings of the present study clearly demonstrate that inhibition of PO by PA is the crucial factor that mediates the effects of *Photorhabdus* infection on both melanotic nodule formation and bacterial virulence. Seo *et al.* [9], showed that the production of metabolites by *Photorhabdus* during a natural infection begins early, with metabolites being detectable in hemolymph extracts as early as 6 h after infection. The authors furthermore showed that melanotic nodule formation in larvae normally occurs within 24 h of infection by bacteria [9].

### 2.3. Insecticidal Activities of PA

**Figure 4:**
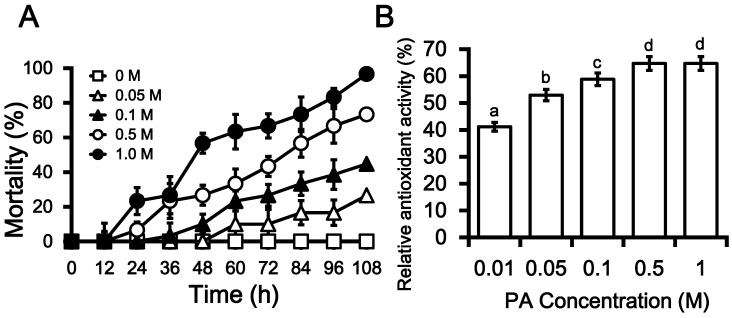
Characteristics of phthalic acid (PA) towards the *Galleria mellonella* larvae in a Dose-dependent course. (**A**) Rate of mortality in *G. mellonella* larvae in the response of the intra-hemocoel injection PA in a way of concentration gradient (0.05 M‒1 M) as compare to the larvae injected with MeOH only, used as control. The resultant values are the means of three replicates (*n* = 3), each with ten larvae per repeat. The error bars represent mean ± SD of the tree repeats; (**B**) Relative antioxidant activity of phthalic acid in does dependent manner (0.01‒1 M) in the reference of Vitamin E, used as positive control. The resultant values are the means of three replicates (*n* = 3). Different letters *i.e*., a, b, c and d over the error bars indicate significant differences at *p <* 0.05 levels as estimated by Duncan’s multiple range test (DMRT). The antioxidant activity was increased with successively higher doses of PA; however, the activity remained the same at 0.5 and 1 M of PA (as indicated by the same letters “d”).

*Photorhabdus* spp. have been extensively studied in terms of their production of extra-cellular compounds that not only contribute to pathogenicity but that can overcome insect immune defenses by suppressing host PO activity [19,30]. To test the insecticidal activity of PA, different concentrations (0.05‒1 M in methanol; MeOH) of the compound were assayed against fifth instar larvae of *G. mellonella* via intra-hemocoel injection. PA toxicity was found to increase with increasing compound concentrations: 108 h after injections, insect mortality was ~40% for 0.1 M PA, while up to 70% and 100% mortality was measured for 0.5 and 1 M PA, respectively (Figure 4A). The IC_50_ value for insecticidal activity of PA was estimated to be 0.25 ± 0.02 M. These results are validated by a study by Seo *et al*. [9] in which compounds (indole, oxindole, and *p*-hydroxyphenyl propionic acid) identified in a culture extract of *P. temperata* were found to cause 100% mortality in insects. The insecticidal activities of 36 PA derivatives against *Plutella xylostella* larvae were evaluated by Feng *et al*. [31], and it was found that almost all the derivatives that were assessed are deadly to the insect, causing 100% mortality in the larvae. In addition they concluded that although the PA derivatives were highly active against a broad spectrum of lepidopterous insects, yet these derivatives posed low acute toxicity towards the mammals and therefore, they were thought to be non-hazardous to the environment [31]. The findings of an investigation into the toxicity of PA in insects by Mayer *et al*. [32] further reinforces our findings that PA has insecticidal capacity. A number of chemical and biochemical approaches have been adopted to obtain new range of insecticides against the insects, already immune for the available insecticides [31]. Safe usage of PA and its derivatives, as insecticidal agent is a point of interest for upcoming studies.

### 2.4. Antioxidant Activity

During the present study, the antioxidant activity of PA was measured in terms of hydrogen-donating or radical-scavenging ability using 2,20-diphenyl-1-picrylhydrazyl (DPPH) as a stable radical [33,34]. Vitamin E was used as positive control, and the antioxidant activity of PA was measured relative to the positive control. PA was found to have dose-dependent DPPH-scavenging activity and the maximum antioxidant activity of PA (64.7%) was observed for 0.5 M PA, beyond which no further increase in antioxidant activity was observed (Figure 4B). Antioxidant activity was shown to increase with increasing PA concentrations, except in the case of the highest concentration tested (1 M), for which no further increases were observed even up to 1.5 h later.

Antioxidant, antibacterial secondary metabolites such as cinnamic acid and its derivatives are commonly produced by *Photorhabdus* spp. [35], and it is hypothesized that during the attack of an insect by the infective juvenile (IJ), the IJ faces an environment of stress which triggers the release of antioxidants to mediate the unfavorable conditions and thus to neutralize the reaction [6].

The antioxidant activity of PA described here is in accordance with a previous report which identifies PA as one of the key components of plant extracts which exhibit strong antioxidant and antibacterial activities [33], confirming the characterization of PA as an antioxidant in this study. The suggestion that compounds isolated and purified from *Photorhabdus* spp. possess dual-activity (antibiotic and antioxidant activities) further supports the characterization of PA purified from *P. temperata* M1021 as an antioxidant and antimicrobial compound.

### 2.5. Antimicrobial Activity

*Photorhabdus* bacteria secrete a versatile armory of molecules that exert a range of antimicrobial activities, thereby minimizing competition from non-symbiotic bacteria and preventing microbial putrefaction of nematode-infected insect cadavers [6,10]. The antibacterial activity of the PA isolated from *P. temperata* M1201 in this study was evaluated in five test bacterial strains, including three Gram-negative bacteria and two Gram-positive bacteria. Antibacterial activity was evaluated using a colorimetric 2,3,5-triphenyltetrazolium chloride TTC assay. The antibacterial activity of PA (0.01‒1 M) was assessed quantitatively by determining the percentage inhibition of bacterial growth relative to the growth of negative control (MeOH)-treated bacteria. The antibacterial activities (relative growth inhibition) of the highest concentration (1 M) of PA ranged from 66.4% to 74.4%, measured for *Enterobacter cowanii* Cd-3 and *Pantoea conspicua* Cd-6, respectively (data not shown). Results summarized in Table 1 revealed that the antibacterial potency was assessed by MIC and IC_50_ values however, the inhibitory effect varied according to the type of tested microorganism. The MIC values of PA on test bacterial strains were ranged from 0.1 M to 0.5 M and IC_50_ values from 0.015 M to 0.18 M. Among the bacterial strains, *Citrobacter youngae* RSC-5 was the most sensitive bacterial strain, with IC_50_ value of 0.015 M.

**Table 1:** Antibacterial activities of phthalic acid (PA) assayed using 2,3,5-triphenyltetrazolium chloride (TTC). Antibacterial activity in terms of relative (%) growth inhibition by PA was calculated as a percentage relative to the growth of negative control. The MIC and IC_50_ values of PA on test bacterial strains were calculated from the (%) growth inhibition of bacterial strains. The resultant values of are mean ± SD of three repeats (*n* = 3).

Bacterial Strains	Phthalic Acid Concentration (M)	
	MIC	IC50
***Pantoea conspicua* RSC-6**	0.50	0.08 ± 0.01
***Bacillus aryabhattai* RSC-7**	0.10	0.14 ± 0.01
***Bacillus anthracis* RSC-9**	0.50	0.11 ± 0.02
***Enterobacter cowanii* RSC-3**	0.50	0.18 ± 0.03
***Citrobacter youngae* RSC-5**	0.10	0.015 ± 0.01

*Photorhabdus* spp. have been reported to produce the antimicrobial compounds 2-isopropyl-5-(3-phenyl-oxiranyl)-benzene-1,3-diol and 3,5,-dihydroxy-4-isopropyl-stilbene, as well as the β-lactam compound carbapenem [9,10]. These compounds are thought to prevent infected insect carcasses from putrefying over a period of several weeks [15]. Such antimicrobial compounds present in the culture extract of *Photorhabdus* bacteria have the potential to be exploited for use against a wide range of gram-positive and gram-negative bacteria that pose challenges in medical and agricultural fields [10,13].

These results are in agreement with the findings of a previous study in which PA, as one of the major components of a plant extract, was shown by the disc diffusion method to have antibacterial activity against five clinical isolates of gram-positive (*Staphylococcus aureus*) and gram-negative (*Pseudomonas aeruginosa*, *Salmonella typhi*, *Klebsiella pneumonia*, and *Shigella flexneri*) bacteria [33]. The synthesis of a diverse array of compounds through bioconversion is a common characteristic among *Photorhabdus* spp., and these compounds are predominantly broad spectrum antibacterial compounds which are highly active against a wide range of gram-positive and gram-negative bacteria of medical and agricultural importance [9]. PA is an aromatic compound with known antimicrobial activity and has been detected in plants and microbes, including bacteria; however, PA has not previously been identified in *Photorhabdus* spp. in the present study PA is identified from *Photorhabdus*, however more detail study is needed to evaluate it for the agriculture use.

## 3. Experimental

### 3.1. Insect and Bacterial Growth Conditions 

The *Photorhabdus temperata* M1021 strain identified and characterized from soil entomopathogenic nematodes collected from the South Korean locations described in our previous study (KACC accession number 91627P) was used in the present study [7]. *P. temperata* bacterial cultures were routinely maintained in Luria-Bertani (LB) broth (0.5% yeast extract, 1% NaCl, 1% tryptone) and incubated at 28 ± 2 °C for 48 h to allow for pre-cultures with initial optical densities (OD_600_) of 0.6 to be prepared. The strain was cultured on LB agar plates and in broth for both short- and long-term storage and subsequent use in experiments.

Five instar larvae of *G. mellonella* were used in bioassays. Larvae were reared from eggs, and were fed an artificial diet according to the procedures described by Ullah *et al.* [2]. Eggs laid on butter paper by wax moths were added into 150 g media and then incubated at 25 ± 2 °C and a relative humidity of 50% ± 5%, which allowed eggs to hatch. The resulting small larvae were then transferred to a larger volume of medium.

### 3.2. Bioassay Guided Fractionation of P. temperata M1021 Extract

*P. temperata* M1021 was cultured in 1000 mL LB media at 28 ± 2 °C in a shaking incubator (200 ± 20 rpm) for 7 days. Seven-day-old cultures were centrifuged at 12,000× *g* for 10 min at 4 °C, and the resulting supernatants were filtered through 0.2 µm cellulose acetate filters (DISMIC; Frisenette ApS, Knebel, Denmark). Culture filtrates (CFs) were acidified to pH 2.8 ± 0.2 with 1 N HCl, after which they were extracted three times with two volumes of EtOAc. The organic phases were combined and evaporated under vacuum at 40 °C using a rotary evaporator (Sunil Eyela, Seongnam, Republic of Korea). Almost-dry residues were resuspended in 20 mL 100% MeOH and were used in this form for initial bioassays against *G. mellonella* larvae. The insecticidal bioassays were performed by injecting 10 µL extract into the hemocoels of fifth instar *G. mellonella* larvae using a 10 µL Hamilton syringe (Hamilton Co, Reno, NV, USA). EtOAc alone and a culture extract of *E. coli* DH5α, processed in the same way as those of *P. temperate*, were used as negative controls. Ten insects were used per treatment and the experiment was carried out in triplicate. Moreover, the bioactive extract was used for subsequent fractionation through bioactivity-guided fractionation procedure, followed for metabolite purification. The EtOAc extract (3.46 g) dissolved in 20 mL 100% MeOH was absorbed onto silica and subjected to fractionation by flash chromatography on a C_18_ column (Luna 5 μm, 100 Å, 250 × 4.60 mm; Phenomenex, Torrence, CA, USA) filled with silica gel (70–230 mesh; Merck, Darmstadt, Germany) using a gradient of EtOAc-hexane (10:90, 50:50, and 100:0; *v*/*v*) and MeOH:EtOAc (2:98, 5:95, 10:90, 20:80, 50:50, and 100:00; *v*/*v*). Thin-layer chromatography (TLC) revealed the MeOH:EtOAc 100:00 fraction to contain compound **1**.

#### Reverse-Phase High-Performance Liquid Chromatography Analysis

The bioactive fraction was further purified by high-performance liquid chromatography (HPLC). The sample (10 µL) was injected into an HPLC instrument (Varian, Inc., Palo Alto, CA, USA) equipped with a C_18_ column using the following setup and conditions: (1) Shimadzu CBM-10 coupled with UV-VIS detector (SPD-10A) with pumps A and B (LC-10AD); (2) Solvent A, 100% MeOH; Solvent B, 5% acetic acid in water; (3) Solvent program: 0–20 min: 50% A, 50% B; 20–40 min: 80% A, 20% B; 40–60 min: 100% A, 0% B; (4) 1 min/mL flow rate; (5) C_18_ column (Luna 5 µm; 100 Å; 250 × 4.60 mm); (6) Single injections of 20 µL with a 100 mg fraction.

### 3.3. GC-MS and NMR Analyses

The purified compound **1** was further analyzed by single ion monitoring GC-MS (6890N network GC system and a 5973N network mass selective detector; Agilent Technologies, Palo Alto, CA, USA) using an HP-5MS column (30 m × 0.25 mm [i.d.], 0.25 µm film thickness). Helium was used as a carrier gas at a constant flow rate of 1.0 mL/min. Purified fraction (2 μL) was injected into the column in a split ratio of 1:20 for analysis with ionization energy of 70 eV. The helium carrier gas was maintained at a head pressure of 30 kPa and the oven was programmed to have a starting temperature of 60 °C for 3 min, after which the temperature was maintained at 300 °C for 48 min. The mass detector was operated in electron impact mode with an ionization energy of 70 eV, a scanning range of 33–550 atomic mass units (amu), and a scan rate of 1.4 scans/s. Purified samples were identified by comparing mass spectra and retention indices in the spectral database. The NMR spectrum of 1,2-benzenedicarboxylic acid (PA) was obtained using an NMR spectrometer (Advance Digital, Bruker, MA, USA) operating at 500 MHz (^1^H, ^13^C).

### 3.4. Assessment of PO and Nodule Inhibitions by PA

Hemolymph PO inhibition activities of different concentrations of PA (0.05‒1 M dissolved in MeOH) were determined using a L-3,4-dihydroxyphenylalanine (L-DOPA; Sigma-Aldrich, St. Louis, MO, USA) substrate-based assay carried out in a microplate. Fifth instar larvae of *G. mellonella* were placed at −20 °C for 10 min, surface sterilized with 70% EtOH, cut at the abdominal proleg, and bled into a pre-chilled sterile polypropylene tube. Hemolymph was then diluted (3:1; *v*/*v*) with 50 mM phosphate-buffered saline (PBS; 0.15 M sodium chloride, 10 mM sodium phosphate buffer, pH 6.5) solution and kept on ice. Hemolymph plasma samples were obtained by centrifugation of hemocytes at 5000× *g* for 10 min 4 °C. PO inhibition assays were carried out in flat-bottom 96-well plates with 100 µL sodium phosphate buffer (50 mM, pH 6.5) containing 2 mM L-DOPA added to 20 µL compound **1** and/or PA and PO activity was monitored by measuring absorbance at 490 nm using a plate reader (Dynatech MR5000, Dynex Technologies, Chantilly, VA, USA) over a period of 1.5 h. The assay was repeated at least three times. It should be noted that this assay detects predominantly dopachrome and/or dopaminechrome rather than melanin itself.

The nodulation assay was performed by injecting 2 × 10^6^ *E. coli* DH5α cells as a positive control into the hemocoels of *G. mellonella* larvae using a microinjector. After 24 h of incubation at room temperature, melanized nodules were counted using a microscope (Leica, Wetzlar, Germany) at a 40× magnification. To test the effect of PA on nodule formation, solutions of PA (0.05, 0.1, 0.5, and 1 M) were prepared in MeOH and 25 µL of each solution was mixed with *E. coli* (2 × 10^6^ cells) before being injected into fifth instar *G. mellonella* larvae. After 24 h of incubation at room temperature, the nodules were counted as above. Ten larvae were used per treatment and each treatment was assessed in triplicate.

### 3.5. Insecticidal Bioassay

The insecticidal activity of PA (0.05–1 M) was assayed by intra-hemocoel injection of PA into the hemocoels of *G. mellonella* larvae. A 10 μL Hamilton syringe was used to inject 10 μL test sample into the larvae. The injected larvae were transferred into a 90 mm petri dish, incubated at 25 ± 2 °C and a relative humidity of 50%, and mortality rates of larvae were evaluated for 120 h. Larval survival was scored daily, where larvae were considered dead by an absence of movement in response to touch. Larvae injected with 10 μL MeOH were used as control larvae to account for death due to physical trauma from intra-hemocoel injection. Ten larvae were used per treatment; the assays were carried out in triplicate, and all experiments were repeated at least three times.

### 3.6. Antioxidant Activity

The antioxidant activity of PA was measured in terms of hydrogen-donating or radical-scavenging ability using DPPH as a stable radical. Scavenging of DPPH indicates free radical-reducing activity of extracts based on a one-electron reduction. Different concentrations of PA (0.01–1 M) were prepared in MeOH and 0.1 mL of each solution was added to 3.9 mL freshly prepared DPPH (0.1 mM in MeOH). Samples were incubated for 1.5 h at room temperature in the dark, after which absorbance was measured at 517 nm using a spectrophotometer (Shimadzu, UV-1800, Kyoto, Japan). MeOH was used as blank and vitamin E was included as a positive control. The antioxidant index (AI) was calculated using the formula [(*A*_C_ (0) − *A*_A_ (*t*))/*A*_C_ (0)], where *A*_C_ (0) is the absorbance of the control at *t* = 0 min and *A*_A_ (t) is the absorbance of the antioxidant at *t* = 30 min. Tests were carried out in triplicate (*n* = 3).

### 3.7. Antibacterial Activity Assay

Five bacteria, including three Gram-negative (*Pantoea conspicua* RSC-6, *Enterobacter cowanii* RSC-3, and *Citrobacter youngae* RSC-5) and two Gram-positive (*Bacillus aryabhattai* RSC-7 and *Bacillus anthracis* RSC-9) strains, were selected for antibacterial activity assessments. Bacterial strains were grown in LB broth (5 g/L yeast extract, 10 g/L peptone, 5 g/L NaCl, pH 7.0) overnight at 28 ± 2 °C, after which the bacterial suspension was diluted (1 × 10^6^ cfu/mL) for detection. The chromogenic reagent (TTC) used to assess the antibacterial activity of PA was dissolved in sterile distilled water (5 mg/mL) at room temperature and filtered through a 0.22 μm filter. A range of PA concentrations (0.01–1 M) was assessed and test sample solutions (10 μL) were added to dilute bacterial suspensions (90 μL) in the wells of a 96-well microplate. Negative control wells contained 90 μL inoculum and 10 μL MeOH, while streptomycin sulfate (Sigma-Aldrich) was used as a positive control. After the plates were agitated using a plate shaker to mix the contents of the wells and incubated in the dark at 28 °C for 3 h, 10 μL TTC was added into each well and the plate was incubated for 30 min. To determine the IC_50_ value of PA for each bacterial strain, the microplate incubated with TTC was centrifuged at 10,000× *g* for 5 min. Supernatants were removed, and 200 μL EtOH (50%; *v*/*v*) was added to each pellet to extract the colored formazan products from the cells. The absorbance of each sample was measured at 510 nm using a microplate spectrophotometer. Only living microorganisms can convert TTC to formazan to yield the red color which can be measured spectrophotometrically and the percentage (%) bacterial growth inhibition was thus determined by the formula [(*A*c − *A*t)/*A*c] × 100, where *A*c represents the average of triplicate absorbance values of the negative control (MeOH) samples and *A*t represents the average of triplicate absorbance values of the test samples. Relative (%) antimicrobial activity values were expressed as mean ± standard deviation of three independent experiments (*n* = 3).

### 3.8. Statistical Analysis

Means and standard deviations of data were determined using Microsoft Office Excel software (Microsoft; version 2013). Mean values were assessed by the Duncan’s multiple range test using a *p* value of 0.05 (Analysis of variance; SAS release 9.1; SAS, Cary, NC, USA).

## 4. Conclusions

The bioactive compound **1** extracted from *P. temperata* M1021 exhibited PO inhibition in *G. mellonella* larvae, and thus, was subjected to identification and characterization analyses (GC-MS and NMR). Compound **1** was identified as phthalic acid (PA) and showed significant toxicity toward *G. mellonella* larvae as well as inhibitory effects on the insect’s immune responses as determined by assessment of hemocyte nodulation in the larvae guts. PA was furthermore, found to have antibacterial and antioxidant properties, suggesting that the biosynthesis of this compound in *P. temperata* M1021, could be a factor of insecticidal activities along with many other proteinous and non-proteinous toxins.

## Supplementary Materials

Supplementary materials can be accessed at: http://www.mdpi.com/1420-3049/19/12/20913/s1.
