# Supplementary material for: An Insecticidal Compound Produced by an Insect-Pathogenic Bacterium Suppresses Host Defenses through Phenoloxidase Inhibition
Source: Molecules. 2014 Dec 12;19(12):20913–28. doi: 10.3390/molecules191220913 (PMC6271226; doi:10.3390/molecules191220913)

Supplementary Materials

Figure S1. A schematic presentation of the compound purification from *Photorhabdus temperata* M1021. (**A**) A flow sheet the process followed during the solvent-solvent extraction. Finally the M1 compound obtained from the MeOH/EtOAc (100:0 v/v) was subjected to thin-layer (TLC) chromatography. (**B**) TLC plates of fractions M 1 to M 11 were run on TLC plates and visualized under 254 nm.

A

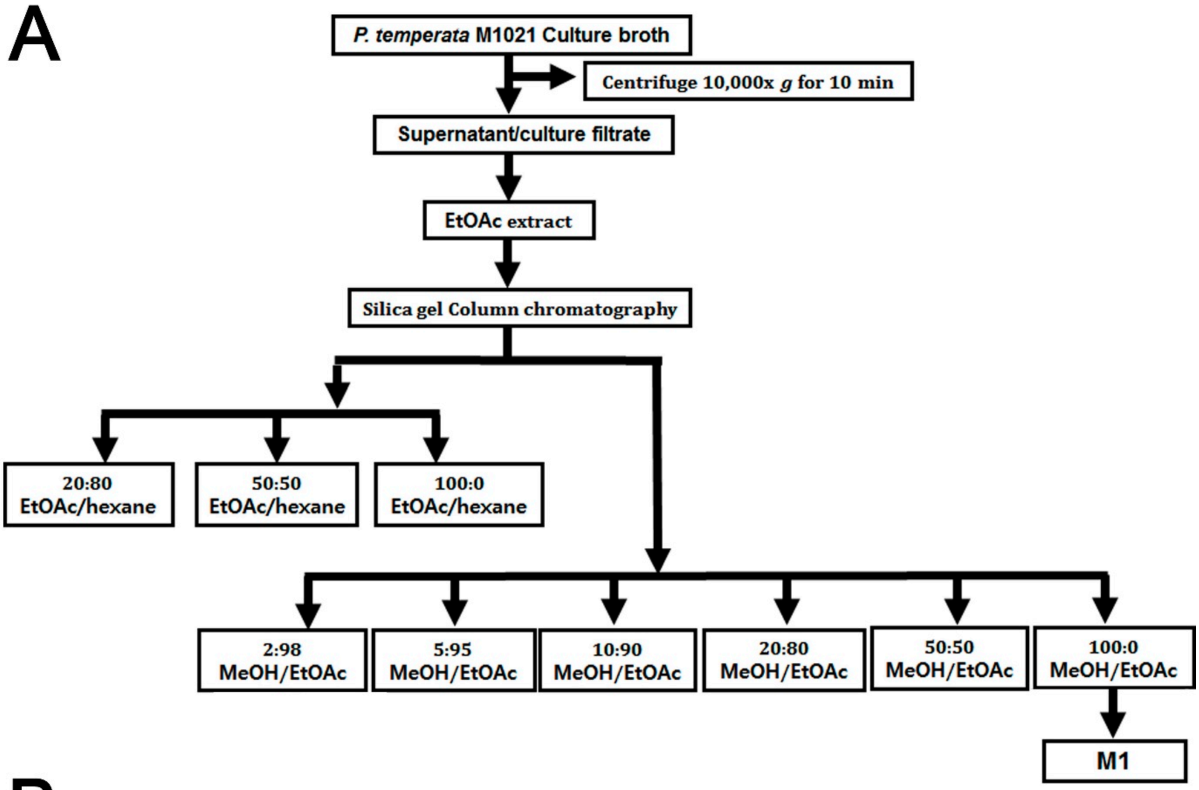

B

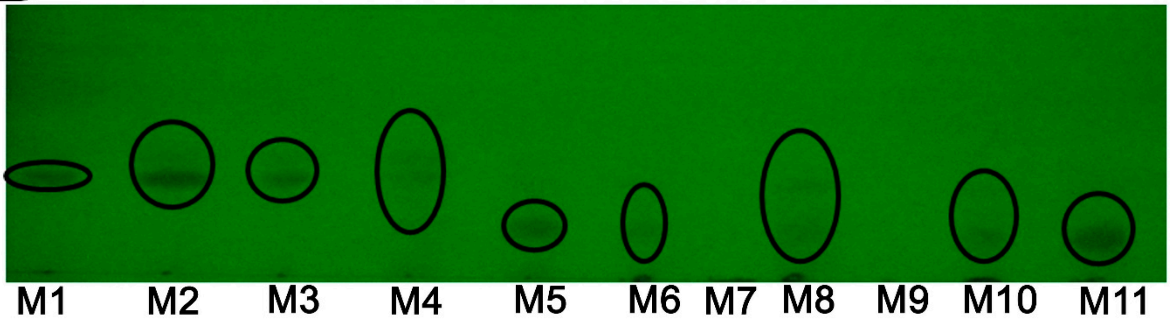

Figure S2. Proton NMR (^{1}H-NMR) at 500 MHz of the compound **1**. The compound was purified from *Photorhabdus temperata* M1021 through different chromatographic techniques. Finally the compound was identified through ^{1}H-, and ^{13}C-NMR.

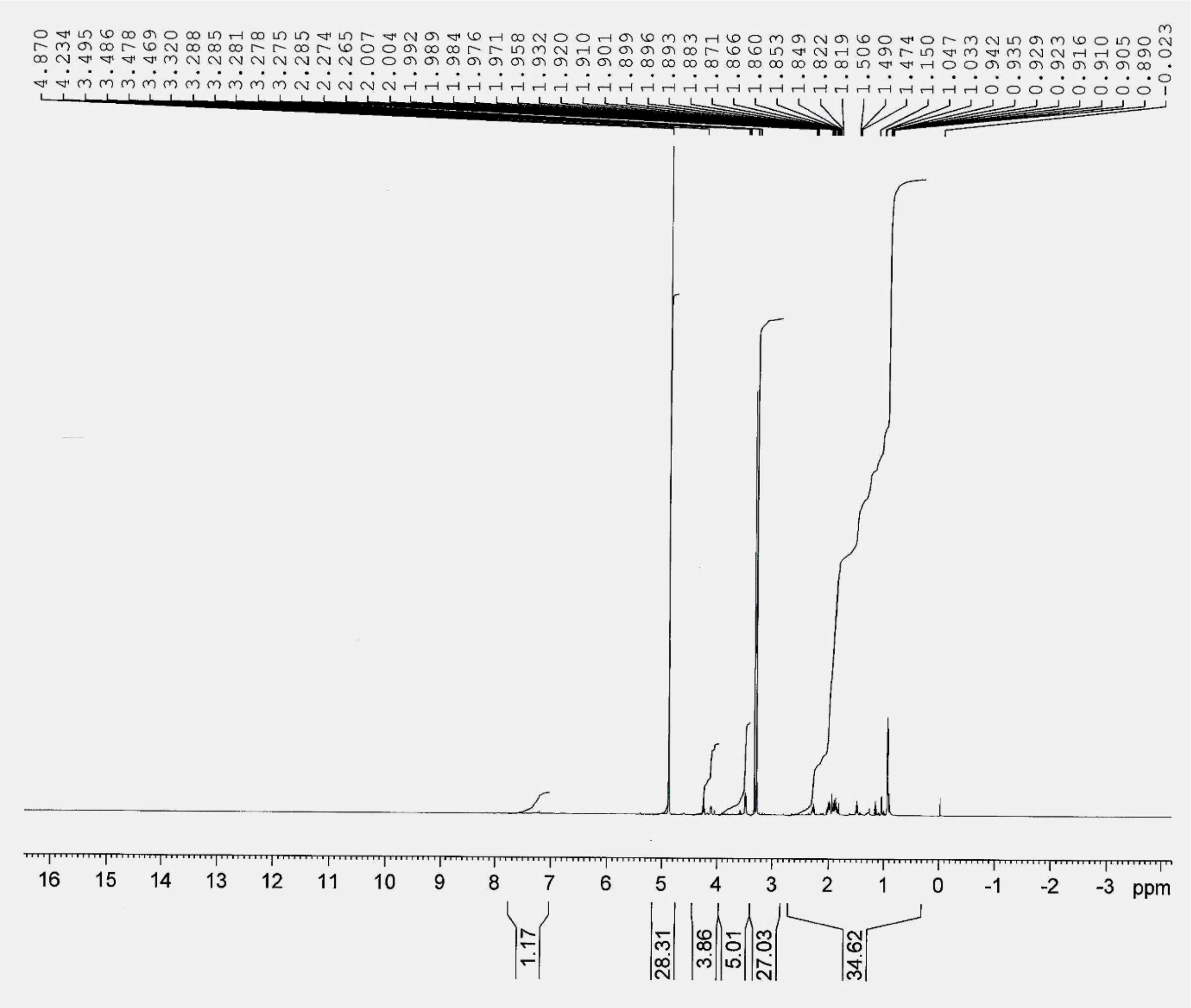

Figure S3. Carbon NMR (^{13}C-NMR) at 500 MHz of the compound **1**. The compound was purified from *Photorhabdus temperata* M1021 through different chromatographic techniques. Finally the compound was identified through ^{1}H-, and ^{13}C-NMR.

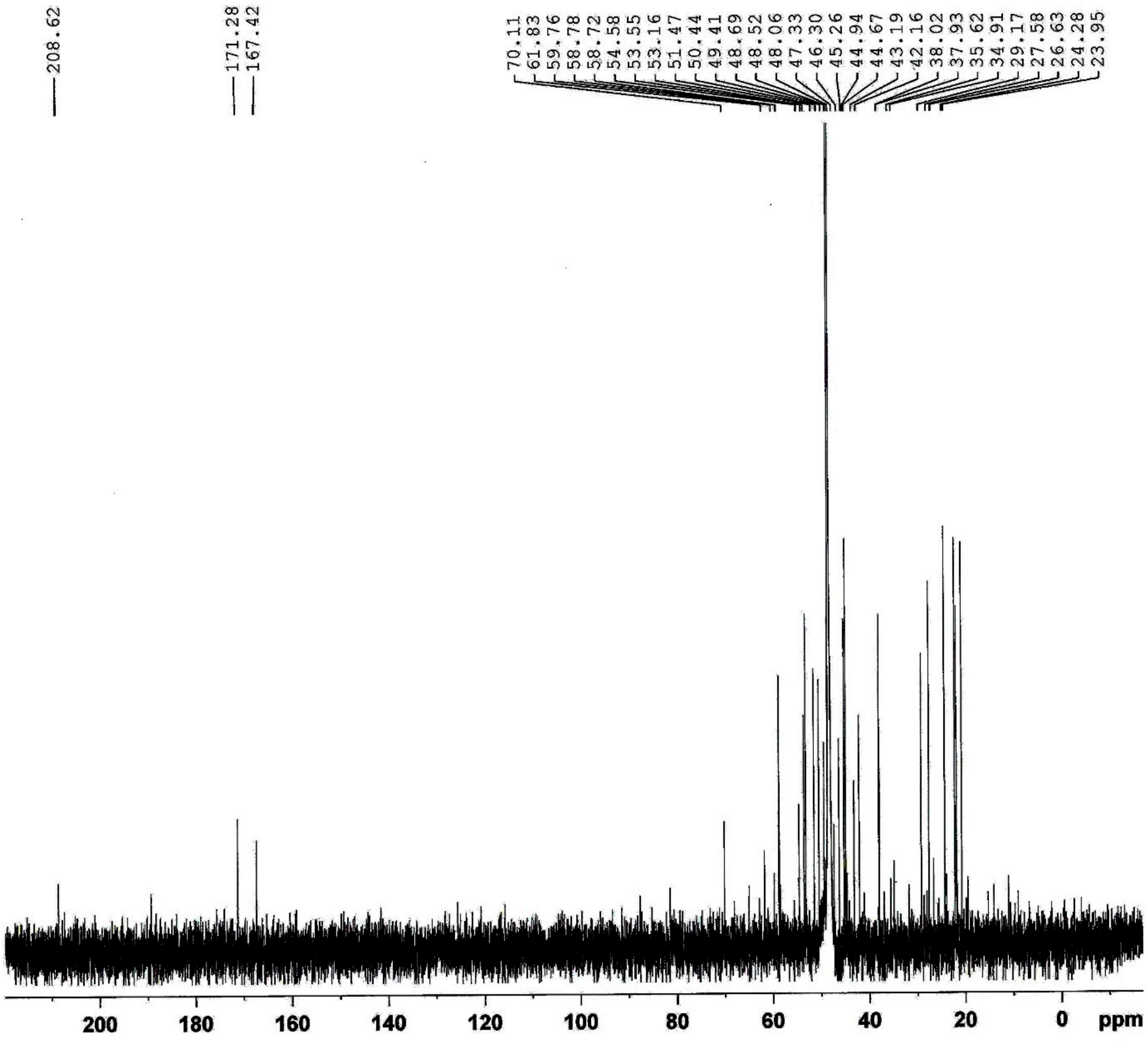

Supplement: Supplementary file 1 [file molecules-19-20913-s001.pdf]
